# Supplementary material for: “The system is a bit broken…” a qualitative exploration of barriers in the pathway for diagnosing Developmental Coordination Disorder
Source: PLoS One. 2026 Mar 3;21(3):e0343972. doi: 10.1371/journal.pone.0343972 (PMC12956065; doi:10.1371/journal.pone.0343972)
Supplement: S1 Appendix — (DOCX) [file pone.0343972.s001.docx]

Supporting Information 1 - Interview Agendas

**General Practitioner**:

1. Could you give us a brief overview of what Developmental Coordination Disorder is and how it presents?
2. Could you give us a brief overview of what Dyspraxia is and how it presents?
3. Could you give us a brief overview of what Developmental Disorder of Motor Function is and how it presents?
4. What is the process that you follow when a child presents with motor difficulties?

Prompts:

For example -

- - Physical tests;
  - Questions to parents;
  - How long is spent in such consultations?
  - Do you access previous records to establish if there is anything else going on?

1. What is the threshold for referrals?
2. What underpins this threshold being the threshold?
3. If a child is borderline; do you refer? Can you explain why?
4. Are there any resources for the families of those children who are referred? What are these?
5. Are there any resources for the families of children who aren’t referred? What are these?
6. What has the potential to impact your ability to assess and support?

Prompts:

- - Staffing;
  - Time;
  - Resources;
  - Waiting lists?

**Paediatrician**:

1. Could you give us a brief overview of what Developmental Coordination Disorder is and how it presents?
2. Could you give us a brief overview of what Dyspraxia is and how it presents?
3. Could you give us a brief overview of what Developmental Disorder of Motor Function is and how it presents?
4. What is the process that you follow when a child presents with motor difficulties?

Prompts:

For example -

- - Physical tests;
  - Questions to parents;
  - How long is spent in such consultations?
  - Do you access previous records to establish if there is anything else going on?

1. What is the threshold for referral to OT / PT – and how do you distinguish between the two?
2. What underpins this threshold being the threshold?
3. If a child is borderline; do you refer? Can you explain why?
4. Are there any resources for the families of those children who are referred? What are these?
5. Are there any resources for the families of children who aren’t referred? What are these?

PART 2 – back from OT/PT

1. What information influences the decision to make a diagnosis?
2. If a child is not diagnosed with DCD; could they receive an alternative diagnosis? If so what could this be?
3. How do you use the information that comes to you from OT/PT in diagnoses?
   - We ask as there have only been 154 diagnoses in the past 40 years; how do you make sense of this?
4. What has the potential to impact your ability to diagnose and support?

Prompts:

- - Staffing;
  - Time;
  - Resources;
  - Waiting lists?

**Occupational Therapy and Physiotherapy**

1. Could you give us a brief overview of what Developmental Coordination Disorder is and how it presents?
2. Could you give us a brief overview of what Dyspraxia is and how it presents?
3. Could you give us a brief overview of what Developmental Disorder of Motor Function is and how it presents?
4. Could you tell us what the defining differences are between the three?
5. What is the process that you follow when a child presents with motor difficulties? Pre and post FUNMOVES

Prompts:

- - Physical tests;
  - Questions to parents;
  - How long is spent in such consultations?
  - Do you access previous records to establish if there is anything else going on?

1. What is the threshold for recommending diagnosis via Paediatrics?
2. What underpins this threshold being the threshold?
3. If a child is borderline; do you recommend diagnosis? Can you explain why?
4. Are there any resources for the families of those children who fall below 5th percentile on MABC? What are these? Pre and post FUNMOVES
5. Are there any resources for the families of children who don’t fall below 5th percentile on MABC? What are these? Pre and post FUNMOVES
6. What resources do you use? Pre and post FUNMOVES
7. What kind of support is provided for those with a diagnosis?
8. What kind of support is provided for those who don’t have a diagnosis?
9. If you consider the children referred to you; what percentage of those children have diagnosable motor and coordination difficulties?
10. What has the potential to impact your ability to assess and support? Pre and post FUNMOVES*

Prompts:

- - Staffing;
  - Time;
  - Resources;
  - Waiting lists?

1. What are your thoughts on FUNMOVES?

**FUNMOVES is a universal screening and intervention resource that was adopted by OT and physio services in the district in 2023.*

**School Nurse / Special Educational Needs and Disabilities Coordinator / Physical Development Team / Educational Psychologist**

1. Could you give us a brief overview of what Developmental Coordination Disorder is and how it presents?
2. What do you look for when you suspect a child has motor skill difficulties?
3. What is the process that you follow when you identify a child with suspected motor difficulties?

Prompts:

For example -

- - Physical tests;
  - Questions to parents;
  - Do you access previous records (e.g. ECHP) to establish if there is anything else going on?
    - Resources

1. What are referral pathway options for you as a [role]?
2. What is the threshold for your referrals?
3. What underpins this threshold being the threshold?
4. Are there any resources for the families of those children you refer? What are these?
5. How do you communicate potential motor skill difficulties to parents?
6. What has the potential to impact your ability to assess and support?

Prompts:

- - Staffing;
  - Time;
  - Resources

**Education Health and Care Plan panel members**

1. Can you tell us about the process of assessment of ECHP applications?

Prompts:

- Who is involved?
- What information do you use?

1. What underpins the decision to accept applications?

Prompts:

- - Budgets
  - Hierarchy of need
  - National / local comparison
  - Evidence supplied
  - Pathway (school/ parental)

1. What underpins the decision to reject applications?

Prompts:

- - Budgets
  - Hierarchy of need
  - National / local comparison
  - Evidence supplied
  - Pathway (school/ parental)

1. What proportion of applications are accepted?

Prompts:

- - How does this compare to national?
  - What is missing?
  - Parental vs school based

1. What are the most common diagnoses that underpins EHCP acceptances?

Prompt:

- - Why?

1. Are there any diagnoses / needs that are less likely to be successful at panel?

Prompt:

- - Why?
  - DCD – not getting through.

1. What information do you provide alongside your decision?

Prompts:

- - Justification
  - Resources
  - Information about appeals

1. What is the process for appeal if schools/ parents aren’t happy with the outcome?
2. Generally, how many appeals are successful?

Prompts:

- - Any particular diagnoses/ needs?
